# Gamete donation in the time of DNA surprises

**DOI:** 10.1111/aogs.14483

**Published:** 2022-11-17

**Authors:** Stine W. Adrian, Tine Ravn, Janne Rothmar Herrmann, Randi Sylvest, Minori Kokado, Yukari Semba, Mads Fencker, Anne‐Bine Skytte, Anette Sellmer, Emma Grønbæk, Ayo Wahlberg, Ulrik Kesmodel

**Affiliations:** ^1^ Department of Culture and Learning Aalborg University Aalborg Denmark; ^2^ The Danish Center for Studies in Research and Research Policy Aarhus University Aarhus Denmark; ^3^ Faculty of Law Copenhagen University Copenhagen Denmark; ^4^ Fertility Department Rigshospitalet Copenhagen Denmark; ^5^ Laboratory of Social Sciences Kobe Pharmaceutical University Kobe Japan; ^6^ Institute for Gender Studies, Ochanomizu University Tokio Japan; ^7^ Donor/Donor‐Conceived Person; ^8^ Cryos International Sperm and Egg Bank Arhus Denmark; ^9^ Sellmer Klinik ApS Copenhagen Denmark; ^10^ Donor‐Conceived Person; ^11^ Department of Anthropology Copenhagen University Copenhagen Denmark; ^12^ Department of Obstetrics and Gynecology Aalborg University Hospital Aalborg Denmark; ^13^ Department of Clinical Medicine Aalborg University Aalborg Denmark

The use of gamete donation is expanding worldwide. At the same time, the practices, and perceptions of how donor families are established are changing.

While some countries still mandate anonymous donation to ensure the privacy of the donor and protection of the family unit (eg Spain, China, and Japan), a number of countries have introduced new legislation according to which open‐identity donation is the sole option (eg Sweden, the UK, and Norway).^1^ Additionally, a few countries have enabled donation from both anonymous and open donors, making the recipient responsible for the choice of category instead of the state (eg Denmark, USA, and Iceland).

Given the availability of low‐cost DNA testing and commercial international DNA ancestry sites, anonymity can no longer be guaranteed for future and past donors.^2^ These private DNA‐tests and Ancestry sites base their results on probabilities and claim to be very accurate but are still refining their algorithms. Still, it is unclear what the clinical significance of the test‐results is.

The consequences of these technological developments are as yet unresolved. To address this issue, the ‘Reconfiguring Donor Conception’ network, consisting of social scientists from Japan and Denmark, organized a stakeholder workshop in Copenhagen, in August 2022. Participants included representatives from public and private fertility clinics, gamete donors, parents to donor‐conceived children, donor‐conceived people, private sperm and egg banks, and researchers within the field. Here, we describe the main perceptions and ideas that emerged from this workshop by discussing the following questions: *How do technologies, such as genetic testing and online fora, impact anonymity in donor conception? What challenges or possibilities emerge in relation to donor conception as new technologies are used? Are there ways to organize donor conception more responsibly in the future?*

Empirical evidence suggests that what is central to the well‐being of offspring is not whether the donor is anonymous but whether parents disclose the donor conception to their child early in life. Positive associations between early disclosure and feelings about donor conception have been found.^3^, ^4^ At the same time, the matter of donor disclosure has been a persistent matter of concern within the psychosocial literature, as it may give rise to parental dilemmas^5^ and may not always be associated with positive outcomes for all parties involved.^6^, ^7^, ^8^

Presently, both legislations and expectations regarding donor disclosure and anonymity are rapidly changing. For example, in Denmark, it was only in 2012 that legislation enabled the use of open donors. Furthermore, while medical staff today will support a recipient's decision to disclose how their child has been conceived, heterosexual couples were guided to keep donor conception a secret up until the 2000s.^9^

The changing morality and status of the donor often place donor families in challenging situations, in which regrets and contradictory perceptions of the donation may emerge together with conflicting perceptions of whose rights and interests should be safeguarded. This is especially likely to come about when a donor‐conceived person is told of their conception late in life, when a donor‐conceived person feels a strong need to identify the donor, or when parents regret the choice of anonymous donation, but at the time of conception had only this option. The changing norms surrounding donor conception have been further accelerated by direct‐to‐consumer genetic testing, as donor‐conceived people may learn of their conception as a *DNA surprise*, an unexpected finding arising from a curiosity about one's ancestry and/or genetic make‐up. This is not only the case for donor‐conceived people. Donors' relatives may also face DNA surprises if the donor kept their donor status a secret. Often in such cases, those involved in the donation, including the donor's relatives, do not necessarily have anywhere to go for needed support.

A number of online fora and registers have been established because many parents, donor‐conceived people, and donors are faced with questions about how to be a donor family and wish to learn more about it or potentially connect with genetic relatives. However, not everyone feels safe sharing personal information on Facebook or unofficial, unregulated registries.

At the workshop stakeholders did not agree on questions such as anonymity, or whether the number of donor offspring should be limited and to what extent. Instead, a consensus emerged around the idea of developing a more comprehensive support system to help donor families, including donor relatives to receive help as challenges emerge. A number of solutions were suggested.

Firstly, support could be provided through an independent website. Such a website could provide information and stories regarding the lived experiences of donor‐conceived families, including the types of challenges that may emerge and how they can be overcome. Moreover, such a platform could provide information on current legal rights and policies related to donor conception.

Stakeholders also strongly emphasized the need for professional counseling services catering to the various needs and perceptions and complex situations that unfold over time within donor‐conceived families. Some women may for example regret that they had transnational treatment in a country where they used an anonymous donor, living in a country where only open donation is available. In that case neither she, nor the child, would have any rights to donor information. The only way to potentially obtain information would be (as many do) to breach anonymity by using donor registers and DNA‐tests. This might result in finding genetic relatives which can prove to be a positive experience. However, for some the number of genetic relatives can also be overwhelming. Others fear the risk of consanguinity, or they question what types of relations can be established with a donor, and with his or her other genetic relatives. Central to these considerations is the open question: What is the role of genes in building relations, social networks, and families? Relatedly, what type of relations can parents, donors, and donor‐conceived people expect to establish based on shared DNA?

These questions also serve as a call for an independent international and flexible registry that could enable different levels of contact between genetic relatives, including donors who wish to lift their anonymity.

Overall, the workshop revealed that gamete donation can and must be organized in a way that supports those involved in donation over time as new moralities and challenges emerge. The need to rethink how this is done has become ever more emergent with the increasing availability of low‐cost DNA tests and online fora. At the same time, the workshop raised the questions of who should be responsible for providing and developing an independent support website; who should provide counseling programs to the parties involved in sperm donation; and who should develop, update, and keep a donor registry for gametes that are marketed and sold on the international market.

The increased availability and use of third‐party reproduction and consumer genetic testing speak to transformations that are inherently global in nature. Therefore, solutions to address the accompanying ethical, regulatory, and practical challenges and unexpected consequences must yield cross‐country answers. While particular supranational, state‐level, and public and private actor responsibilities are not easily delegated, it is evident that the provision of lifelong support is necessary to those families involved in gamete donation. This demands a collective effort, including funding for further international research to understand how to develop better support for donor families, as well as setting up a comprehensive support system globally. The aforementioned questions represent a first step toward conducting donor conception more responsibly in the future, arising from collective stakeholder deliberation on experienced challenges. Considering the number of families involved in donor conception, it is about time that we start rethinking what donation means for families and donors and how it shapes families today.
